# A new marker of primary care utilization - annual accumulated duration of time of visits

**DOI:** 10.1186/s13584-017-0159-y

**Published:** 2017-08-10

**Authors:** Talya A. Nathan, Arnon D. Cohen, Shlomo Vinker

**Affiliations:** 1 The Department of Family Medicine, Sackler School of Medicine, Tel Aviv University, Tel Aviv, Israel; 2 Clalit Health Services, Tel Aviv; 3) Medical Division, Leumit Health Services, Tel Aviv, Israel

**Keywords:** Primary care visit/consultation, Family physician, Visit/consultation duration

## Abstract

**Background:**

Most of the research on primary care workload has focused on the number of visits or the average duration of visits to a primary care physician (PCP) and their effect on the quality of medical care. However, the accumulated annual visit duration has yet to be examined. This measure could also have implications for the allocation of resources among health plans and across regions. In this study we aimed to define and characterize the concept of "Accumulated Annual Duration of Time" (AADT) spent with a PCP.

**Method:**

A cross-sectional study based on a national random sample of 77,247 adults aged 20 and over. The study’s variables included annual number of visits and AADT with a PCP, demographic characteristics and chronic diseases. The time period was the entire year of 2012.

**Results:**

For patients older than 20 years, the average annual number of visits to a PCP was 8.8 ± 9.1, and the median 6 ± 10 IQR (Interquartile Range). The mean AADT was 65.8 ± 75.7 min, and the median AADT was 43 ± 75 IQR minutes. The main characteristics of patients with a higher annual number of visits and a higher AADT with a PCP were: female, older in age, a higher Charlson index and a low socio-economic status. Chronic diseases were also found to increase the number of annual visits to a PCP as well as the AADT, patients with chronic heart failure had highest AADT in comparison to others (23.1 ± 15.5 vs. 8.6 ± 8.9 visits; and 165.3 ± 128.8 vs. 64.5 ± 74 min). It was also found that the relationship between AADT and age was very similar to the relationship between visits and age.

**Conclusion:**

While facing the ongoing increase in a PCP’s work load and shortening of visit length, the concept of AADT provides a new measure to compare between different healthcare systems that allocate different time frames for a single primary care visit. For Israel, the analysis of the AADT data provides support for continued use of the number of visits in the capitation formula, as a reliable and readily-accessible indicator of primary care usage.

**Electronic supplementary material:**

The online version of this article (doi:10.1186/s13584-017-0159-y) contains supplementary material, which is available to authorized users.

## How this fits in

Novel concept of "Accumulated Annual Duration of Time" spent with a primary care physician as a new measure to assess health services.

- We present a new measure "The accumulated annual visit duration" with primary care physicians that had not been evaluated in the literature.
- Our findings support cumulative duration as a parallel mean to the number of visits for health services assessment. This novel concept may serve as a new standardized comparative measure to evaluate and unify the characteristics of high quality primary care.
- New primary care guidelines should also refer to the optimal amount of time needed to be spent on health topics within the visit, rather than focusing on the number of visits.

## Background

### Primary care visits

The primary care visit remains the principal opportunity for health care providers to address patient’s needs. The results of the Israel Central Bureau of Statistics (ICBS) for 2009 indicate that the annual average number of visits to the primary care physician (PCP) is 6.2 in the general population of Israel and 16.1 for ages 65 and over. Age and the number of visits of patients with chronic diseases were found to be factors that significantly increase the annual average number of visits [1]. The most recent data found by us suggests that the mean duration of a visit with an Israeli PCP is 10.4 min [2].

The annual average number of visits can vary substantially across countries. One study in the United States calculated a mean of 1.6 PCP (defined as visits to a general practitioner, family physician, pediatrician, geriatrician, or general internist) yearly visits per person as of 2008 [3]. In the WHO European Region, the average outpatient contacts per person per year in 2006 was 7.85, and country specific averages for 2006 or the latest available year were 7.0 in Germany, 9.5 in Spain, 5.4 in the United Kingdom, 5.7 in the Netherlands, 6.6 in Belgium and 11.0 in Switzerland [4].

There is also significant cross-country variation in visit duration. In the United States, 2006 data from the Centers for Disease Control and Prevention (CDC) found that the mean duration of face-to-face visits with PCPs (general or family practice) was 19.5 min [5]. In Europe, it was found that the mean length of a visit with a PCP (general practitioner) was 7.6 min in Germany, 7.8 min in Spain, 9.4 min in the United Kingdom, 10.2 min in the Netherlands, 15.0 min in Belgium and 15.6 min in Switzerland [6]. A study by Bindman et al. found in a 2001–2 cross-sectional analysis that the average duration of a face-to-face visit with a PCP in the US (general internists, general pediatricians, and family practitioners) was 16.5 min, about 10% longer than with general practitioners in Australia (14.9 min) and New Zealand (15 min). Visit lengths were longer in the US for all age and gender groups. Because the average number of primary care visits per capita was greater in New Zealand and Australia, however, the mean per capita annual exposure to primary care physicians in the US (29.7 min) was about half of that in New Zealand (55.5 min) and about a third of that in Australia (83.4 min) [7].

Studies from various countries have found that the length of an ambulatory visit with PCPs is influenced by increasing age, presence of psychosocial problems [8], gender (women) and greater number of new problems discussed in the visit [6].

#### Visit duration and patient outcomes

Research in the matter has shown that longer PCP visits were associated with a range of better patient outcomes [9, 10], including more statements about health education and prevention [11], as well as higher rates of preventive medical measures such as vaccinations [12, 13], and mammography referrals [14]. The duration of PCP care was also associated with lower costs of inpatient and outpatient care and with a lower risk of hospitalizations [15]. Wilson et al. first concluded that a PCP with a higher average visit length is more likely to provide visits that include important aspects of care, and that longer visit length can therefore be used as a quality indicator [16]. They later conducted a systemic review, which found that in interventional studies that had been performed by altering same physicians’ visit length the above mentioned effect had not been demonstrated. However, their findings were not sufficient to support or resist a policy of altering PCP visit length, and due to many limitations of the study, it was difficult for them to define length as a marker of quality of care [17].

When analyzing the primary care setting, one aspect of the visit is its content. A study by Tai-Seale et al. found that visit length was insensitive to the content of a visit - longer time spent on major topics seemed to have been compensated by limiting the time allocated to minor topics, therefore leaving the visit length more or less the same. Instead, organizational structure, physicians’ practice settings and payment incentives appeared to have more influence on visit length [18]. However, other research suggested that there was a positive association between the number of problems discussed and the mean length of visits. It was found that on average, PCPs spend 11.9 min dealing with 2.5 problems, and a linear relationship was seen at least up to six problems, with the length of visits increasing by an average of 2 min for each additional problem above a baseline of 9 min for the first problem [19]. Abbo et al. found that the number of clinical items addressed during a PCP visit increased from 5.4 in 1997 to 7.1 in 2005, resulting in a decrease in minutes spent per clinical item from 4.4 to 3.8 [20]. Approximately 8% of PCP visit duration was found to be attributable to eight-related conditions included diabetes, hypertension, hyperlipidemia, obesity, cardiovascular disease, osteoarthritis, and low back pain [21]. Chen et al.’s findings suggested that the relationship between quality of care and physician visit duration depends on the type of quality indicator being measured, namely, medication quality indicators vs counseling or screening quality indicators. In their research, they found a clear and consistent relationship between visit duration and provision of counseling and screening-based care [22].

Moreover, nearly one half of a primary care physician’s workday was found to be spent on activities outside the examination room, predominately focused on follow-up and documentation of care for patients not physically present. In the United States, Gottschalk et al. found that national estimates of visit duration overestimate the combination of face-to-face time and time spent on visit-specific work outside the examination room by 41% [23].

However, despite evidence that increasing visit length is more likely to improve primary care, and that longer visit length can therefore be used as a quality indicator, to our knowledge and according to the literature review, we did not find a study that defined the optimal annual accumulated time (complementary to the number of visits) that should be spent with a patient to achieve better quality of care.

#### The potential implications for resource allocation

In many countries, the allocation of financial resources among regions and/or among care providers is based on capitation formulae which try to reflect how the composition of populations served affect the need for health care services. For example, as older people tend to use more health care services, regions and providers serving populations with higher concentrations of the elderly are often given more financial resources per capita. This is done so that they will have enough resources to provide quality care and to eliminate any incentive to avoid caring for elderly persons.

In Israel, for example, when Israel distributes the National Health Insurance monies among health plans, it uses a capitation formula which includes mainly age, gender and other minor affecting parameters. In developing that formula, the government examines how age and gender are related to resource use for the key types of care consumed – hospital care, community services, and medications. As its measure of community service use, the government currently uses the number of physician visits. However, if visit duration varies significantly by age or gender, then the number of physician visits would not be a good indicator of resource use, and AADT would be a more appropriate measure to use. If visit duration does not vary significantly by age or gender then it would make sense to continue to base the capitation formula on the number of visits, as it is easier for the government to collect survey data on the number of visits than on the AADT. When the health plans distribute funds among their regions they also take into account various demographic characteristics (including location) and their relationship to service use. They too face a decision of whether to use the number of visits or AADT in resource allocation decisions, and hence they too have interest in knowing whether visit duration varies by demographic characteristics, as well as by location.

We conducted a cross-sectional study based on the electronic medical records of the largest Health Maintenance Organization (HMO) in Israel to investigate the characteristics of the concept of Accumulated Annual Duration of Time (AADT) that the PCP spends with a patient. This is an important first step towards using AADT in resource planning and allocation, and perhaps even determining the optimum level of AADT.

## Methods

### Population and data source

Data was retrieved from the Clalit Health Services (CHS) central computerized database. CHS is the largest HMO in Israel, covering 54% of the entire Israeli population (about 4,200,000 people in 7 districts). Every person insured by CHS is assigned to a PCP. All the visits to a PCP are fully computerized and the information from the electronic medical records is retrieved to a central repository. The central database includes demographics, information about physician visits, and a register of a selected number of chronic diseases (from the HMO’s registry, diagnosed previously to the visits in question).

The study period was the entire 2012 calendar year. The population of this study consisted of all adult members of the HMO aged 20 and over, from which we draw a national random sample of 83,707. The sampling method was a randomized computer based binary extraction of 2% of all patient data, based on the two last digits of the patients’ social security number.

Of the patients who were randomly selected from the HMO’s database, 1088 died during the study period and 2615 left the HMO. Patients older than age 100 years (*n* = 25), bed-ridden (*n* = 2059) or in a nursing home (*n* = 673) were excluded from the study; therefore, the current analysis included 77,247 patients.

### Data accessed

The number and duration of visits of CHS members with a PCP were retrieved for the study period. Additional patient data included: demographic characteristics: age, gender, country of birth, year of immigration to Israel (Individuals who were born in Ethiopia and immigrated to Israel after 1984 were defined as “new immigrants”. Immigrants from other countries were defined as “new immigrants” if they immigrated after 1990. These represent the two major waves of immigration to Israel that took place in the past 30 years), residency (Large city ≥100,000 citizens, other city, collective settlement - also known as a Kibbutz, cooperative Israeli settlement, small town and non-Jewish settlement), socioeconomic status (SES; low SES was defined as exemption from social security payments); chronic diseases (malignancy, diabetes, hypertension, hyperlipidemia, ischemic heart disease (IHD), chronic heart failure (CHF), status post cerebrovascular accident (s/p CVA), asthma, chronic obstructive pulmonary disease (COPD), dementia, epilepsy, anxiety disorder and drug abuse); and a Charlson comorbidity index [24, 25], which was calculated as well.

The study was approved by the CHS ethics committee at the Meir Medical Center, Kfar Saba, Israel.

### Statistical analysis

Descriptive statistics was the primary method of analyzing the data. The annual number of visits and annual duration of visits (in minutes) were analyzed as continuous parameters. The Central Limit Theorem justifies the results despite the non-normal distribution of these variables.

Demographic characteristics were compared as well as medical characteristics for sub-groups according to number of visits and visit duration, using correlations (for differences between continuous parameters), T-tests (for differences between dichotomized parameters and averages of continuous parameters), chi-squared analysis and Fisher IS (for categorical parameters) and ANOVA (for differences between more than two categories in a parameter). If the ANOVA was found to be significant, a POST HOC analysis using Tukey’s test was performed to distinguish the different categories.

We used multivariate analysis to construct predictive models for comparison between annual number of visits and annual duration of visits.

A Multivariate Linear Regression model was applied to the data to study simultaneously the independent relationship between the demographic (age, gender, SES, residence area, and immigration status) and clinical background (chronic diseases, Charlson comorbidity index) and visit characteristics. The model predicts the probability of higher number of visits and longer annual duration of visits as a function of the explanatory variables. We addressed the non-normal distribution of these variables by using a square root transformation.

A *p*-value of 0.05 or less was considered statistically significant. All results were rounded to tenths (+1 decimal place). All analyses were carried out with the assistance of The Statistical Consulting Lab at The School of Mathematical Sciences at Tel Aviv University, using SPSS ver. 21 statistical software.

## Results

Table 1 shows the characteristics of the study population. 52.3% were female and 13.1% were new immigrants. The majority of the study population (81.3%) was between the ages 20–64 (children, up to 20 years old, were excluded from the study), with an average age of 46.5 ± 18.1 years; 41% resided in large cities and only 15.8% were considered to be of low SES. The average Charlson comorbidity index was 3.0 ± 1.1. The average annual number of visits with a PCP during 2012 was 8.8 ± 9.1 visits while the median was 6 ± 10 IQR visits. The average duration of a single visit was 7.6 ± 4.3 min while the median duration was 7 ± 4.5 IQR minutes. The mean annual duration of visits was 65.8 ± 75.8 min while the median annual duration was 43 ± 75 IQR minutes.

**Table 1:** Characteristics of study population and visits with primary care physicians

Characteristic		N (%) (unless stated otherwise)
N		77,247 (100)
Gender	Female	40,434 (52.3)
Age (in years)	Mean (± Range, SD)	46.5 (20–100, 18.1)
	20–64	62,805 (81.3)
	65+	14,442 (18.7)
Charlson comorbidity index (*n* = 77,136)	Mean (± Range, SD)	3.0 (1–6, 1.1)
Socioeconomic status	Low SES	12,185 (15.8)
Country of birth	New immigrants	10,128 (13.1)
Place of residence (*n* = 77,194)	Large city (≥100,000 citizens)	31,642 (41.0)
	Other city	21,970 (28.4)
	Kibbutz (collective settlement)	3018 (3.9)
	Cooperative Israeli settlement	5162 (6.7)
	Small town	6113 (7.9)
	Non-Jewish settlement	9289 (12.0)
Number of visits to a PCP during 2012 (*n* = 77,247)	Mean (± Range, SD) Median (± Range, IQR)	8.8 (0–136, 9.1) 6 (0–136, 10)
	Percentiles	
	25	2.0
	50	6.0
	75	12.0
	90	20.0
AADT (in minutes) spent with a PCP during 2012 (*n* = 77,247)	Mean (± Range, SD) Median (± Range, IQR)	65.8 (0–1659, 75.8) 43 (0–1659, 75)
	Percentiles	
	25	15.0
	50	43.0
	75	90.0
	90	155.0
Duration (in minutes) of a single visit to a PCP during 2012 (*n* = 70,186)	Mean (± Range, SD) Median (± Range, IQR)	7.6 (0–60, 4.3) 7 (0–60, 4.5)

*PCP* Primary Care Physician

*AADT* Accumulated Annual Duration of Time

*SES* Socioeconomic Status

*IQR* Interquartile Range

Table 2 presents the characteristics of the annual number of visits and the annual duration of visits with a PCP during 2012. A positive correlation between the annual number of visits as well as the annual duration of visits was found with both age (0.4) and the Charlson index (0.5). More visits, with a higher AADT were made by women (9.8 ± 9.2 vs. 7.7 ± 8.9 visits and 73.3 ± 76.7 vs. 57.5 ± 73.8 min); by the subgroup of low SES (14.7 ± 11.9 vs. 7.7 ± 8.0 visits and 104.5 ± 98.4 vs. 58.5 ± 68.3 min); and in kibbutzim (11.9 ± 11.9 vs. <8.9 visits and 100.3 ± 116.9 vs. <67.2 min) in comparison to large cities. Those who were new immigrants visited less frequently (7.7 ± 8.1 vs. 9.0 ± 9.2 visits) and had a lower AADT (57.1 ± 67.4 vs. 67.1 ± 76.8 min). Patients with one or more chronic diseases were also found to have made more visits and spent more time with their PCP throughout the year. The most substantial difference was seen among patients with chronic heart failure (CHF) compared to patient without the disease (23.1 ± 15.5 vs. 8.6 ± 8.9 visits, a 167.9% increase and 165.3 ± 128.8 vs. 64.5 ± 74 min, a 156.2% difference) followed by chronic obstructive pulmonary disease (COPD) (20.1 ± 15.1 vs. 8.6 ± 8.8 visits, a 135.3% difference and 143.9 ± 120.9 vs. 63.9 ± 73.4 min, a 125% difference) and hypertension (15.9 ± 11.5 vs. 6.8 ± 7.2 visits, a 133.1% difference and 115.9 ± 98.7 vs. 51.8 ± 61 min, a 123.8% difference).

**Table 2:** Characteristics of the annual number of visits and the Annual Accumulate Duration of Time spent with a primary care physician during 2012

			No. of visits during 2012		AADT during 2012 (in minutes)	
Mean (± Range, SD)			8.8 (0–136, 9.1)		65.8 (0–1659, 75.8)	
Median (± Range, IQR)			6 (0–136, 10)		43 (0–1659, 75)	
Characteristic			Pearson Correlation	*P* Value	Pearson Correlation	*P* Value
Age (in years)			0.4	0.000	0.4	0.000
Charlson comorbidity index			0.5	0.000	0.5	0.000
			Mean (±SD) *T-TEST*	*P* Value	Mean (±SD) *T-TEST*	*P* Value
Gender	Male		7.7 (8.9)	<0.001	57.5 (73.8)	<0.05
	Female		9.8 (9.2)		73.3 (76.7)	
Socioeconomic status	Other		7.7 (8.0)	<0.001	58.5 (68.3)	<0.05
	Low SES		14.7 (11.9)		104.5 (98.4)	
Country of birth	New immigrant		7.7 (8.1)	<0.05	57.1 (67.4)	<0.05
	Other		9.0 (9.2)		67.1 (76.8)	
			Mean (±SD) *ANOVA*	*P* Value	Mean (±SD) *ANOVA*	*P* Value
Place of residence	Large city		8.7 (8.9)	<0.05	66.9 (75.3)	<0.05
	Other city		8.8 (9.0)		62.8 (70.0)	
	Kibbutz		11.9 (11.9)		100.3 (116.9)	
	Cooperative Israeli settlement		8.3 (8.5)		67.1 (78.0)	
	Small town		8.5 (8.8)		62.1 (71.9)	
	Non-Jewish settlement		8.7 (9.5)		59.5 (71.3)	
Chronic Diseases		*N*	Mean (±SD) *T-TEST*	*P* Value	Mean (±SD) *T-TEST*	*P* Value
Malignancy	+	4250	16.4 (12)	<0.05	123.5 (103.4)	<0.05
	−	72,997	8.4 (8.7)		62.4 (72.4)	
Diabetes	+	9065	17.5 (11.9)		124.6 (100.4)	
	−	68,182	7.7 (8)		58 (68.1)	
Hypertension	+	16,848	15.9 (11.5)		115.9 (98.7)	
	−	60,399	6.8 (7.2)		51.8 (61)	
Hyperlipidemia	+	27,266	13.7 (10.7)		100.3 (90.8)	
	−	49,981	6.1 (6.7)		47 (58)	
IHD	+	5604	18.4 (12.6)		132.9 (107.2)	
	−	71,643	8.1 (8.3)		60.5 (70.1)	
CHF	+	985	23.1 (15.5)		165.3 (128.8)	
	−	76,262	8.6 (8.9)		64.5 (74)	
s/p CVA	+	2013	18.7 (13.8)		135.2 (114.3)	
	−	75,234	8.6 (8.8)		63.9 (73.6)	
Asthma	+	4714	12.9 (12.2)		95.2 (97.7)	
	−	72,533	8.6 (8.8)		63.9 (73.7)	
COPD	+	1776	20.1 (15.1)		143.9 (120.9)	
	−	75,471	8.6 (8.8)		63.9 (73.4)	
Dementia	+	605	16.8 (13)		113.3 (98.1)	
	−	76,642	8.8 (9.1)		65.4 (75.4)	
Epilepsy	+	918	13.7 (11.3)		98.1 (94.7)	
	−	76,329	8.8 (9.1)		65.4 (75.4)	
Anxiety Disorder	+	2892	16.6 (12.9)		121.2 (111)	
	−	74,355	8.5 (8.8)		63.6 (73.2)	
Drug Abuse	+	496	11.3 (12.7)		82 (95.6)	
	−	76,751	8.8 (9.1)		65.7 (75.6)	

*AADT* Accumulated Annual Duration of Time

*IQR* Interquartile Range

*SES* Socioeconomic Status

*IHD* Ischemic Heart Disease

*CHF* Chronic Heart Failure

*s/p CVA* status post Cerebrovascular Accident

*COPD* Chronic Obstructive Pulmonary Disease

Table 3 presents data on the average visit duration varied by age and gender, calculated as $\left[\frac{AADT\  during\ 2012}{No.\kern0.5em  of\  visits\  during\ 2012}\right]$ for each age and gender group. The data indicate that visit duration was found to be very similar for both men and women and across age groups.

**Table 3:** Average visit duration in 2012 $\left[\frac{AADT\  during\ 2012}{No.\kern0.5em  of\  visits\  during\ 2012}\right]$, by age and gender

	Average time per visit (in minutes)	
Age group (in years)	Female	Male
20–30	7.8	7.8
30–40	7.4	7.2
40–50	7.1	7.0
50–60	7.4	7.6
60–70	7.4	7.6
70–80	7.4	7.4
80–90	7.3	7.1
90–100	6.8	6.9

Table 4 presents a Multivariate Linear Regression analysis (in square root) for the number of visits (R-squared 0.39) and the AADT (R-squared 0.34) spent with a PCP during 2012. Increase in age was initially associated with a non-linear increase in the number of visits and in the amount of time spent with a PCP, however after age 80 subsequent increases in age showed a decline in the number and duration of visits (See Additional file 1). Women, patients of a low SES and with a higher Charlson index spent more time and paid more visits with their PCP. Being a new immigrant meant fewer and shorter visits, and compared to persons residing in large cities, kibbutz members had the highest visiting rate and spent the most time with their PCP.

**Table 4:** Linear regression (in square root) - number of visits and Annual Accumulate Duration of Time spent with a primary care physician during 2012

		No. of visits during 2012 R-squared 0.39		AADT during 2012 R-squared 0.34	
		Unstandardized Coefficients (B)	*P* Value	Unstandardized Coefficients (B)	*P* Value
Gender (vs. Male)	Female	0.2	0.000	0.5	0.000
Age (in years vs. 20–30)	30–40	0.0	<0.05	0.0	0.999
	40–50	0.2		0.5	<0.05
	50–60	0.5		1.1	
	60–70	0.8		1.8	
	70–80	1.0		2.4	
	80–90	0.9		2.2	
	90–100	0.4		0.7	
Charlson comorbidity index	For every point added	0.6	0.000	1.7	0.000
Socioeconomic status (vs. Other)	Low SES	0.3	0.000	0.7	0.000
Country of birth (vs. Other)	New immigrant	−0.2	0.000	−0.5	0.000
Place of residence (vs. Large city)	Other city	0.0	0.000	−0.1	0.000
	Kibbutz	0.2		0.5	
	Cooperative Israeli settlement	0.0		0.1	
	Small town	0.0		−0.0	0.803
	Non-Jewish settlement	0.0		0.0	0.083

*AADT* Accumulated Annual Duration of Time

*SES* Socioeconomic Status

## Discussion

### Summary

During 2012, the average annual number of visits with a PCP was 8.8 ± 9.1 and the median was 6 ± 10 IQR. The mean AADT was 65.7 ± 75.8 min and the median AADT was 43 ± 75 IQR minutes. The average duration of a single visit was 7.6 ± 4.3 min and the median was 7 ± 4.5 IQR minutes, which is lower than the data known to us prior to this study [2]. This was to be expected following the rise in the PCP’s workload due to population growth and the increase in life expectancy.

The main characteristics of patients with a higher annual number of visits and a higher AADT with a PCP were: female, older in age, a higher Charlson index (all three of which coincide with previously known dat﻿a ﻿[1, 6]), of a low SES (which could be explained by Israel’s public health care system, providing highly available/no cost primary care), and residing in a kibbutz (possibly due to greater accessibility to PCP’s). New immigrants had a lower annual number of visits and a lower AADT with a PCP.

The study also found that average visit duration was very similar for both men and women and across the various age groups. This implies that the relationships of age and gender with the number of visits are similar to their relationships with AADT. Thus, while AADT does a better job of capturing resource use (i.e. the amount of time physicians invest in the care of various types of patients) than does the number of visits, it is reasonable to continue using the number of visits as a proxy for AADT in calculating capitation formulae. In the future, it will be important to examine whether visit duration is also consistent across geographic areas.

### Strengths and limitations

One of the main strengths of the study is that it was based on a national sample from the largest HMO in Israel. Another is its use of thousands of electronic medical records (and not self-reports) from hundreds of general practices. This is in comparison to other studies, where the exposure to primary care was calculated from duration of visits recorded by the physician, and reports on rates of visits to primary care for each country [7, 22, 26, 27]. However, international comparisons may be affected by differences in definitions and in the circumstances in which patients see primary care physicians in different countries. It is possible that some references to outpatient attendances include in part visits with specialists.

Another issue is that there are a substantial number of physician visits that are administrative in nature (repeat prescription, fill out laboratory tests forms, etc.) and do not entail a face-to-face meeting between patient and physician. Although the type of visit is specified in the electronic file, in our experience, this information is usually not accurate and therefore the type of visit is difficult to determine. Therefore, we could not separate between face-to-face and non face-to-face visits, but we believe that they are on the continuum of primary treatment and should be part of the calculated time load on the PCP. Furthermore, some other important limitations exist.

First, an underlying assumption of use of the AADT is that a higher number of annual visits with a shorter average duration are equivalent to a lower number of annual visits with a longer average duration. If the first 2 or 3 min of each visit are used by the physician to greet the patient and look at the electronic notes of past visits, this may not be the case. In addition, these actions may require a minimum time allocated for each visit even when only one problem is raised. These issues are directly related to health care policy planning. Assuming there is a more efficient utilization of physician time with fewer yet longer visits, this aspect requires future examination, which could result in an organizational paradigm shift within the health care system.

Second, we excluded patients that died during the study year. We know that at the end of life the utilization of health care resources can be abundant [28–30], influencing the utilization of primary care visits as well. Therefore, to evaluate this special group, we will need another focused study.

Third, the analysis was not limited to one designated physician per patient, as it was designed to find the importance of the AADT required from primary care as a whole for the treatment of patients. This is an important aspect to be examined in future research, to investigate whether time spent with a patient’s personal primary care physician is more effective.

Another limitation of the current study is a possible information bias - some of those classified as “new immigrants” (as well as others) may live outside Israel. The fact that in recent years new immigrants to Israel usually keep their original residency increases the probability of such events.

### Comparison with existing literature

As expected, chronic diseases were found to increase the number of annual visits with a PCP as well as the AADT. This coincides with previous research, which found patients with multiple chronic diseases having more outpatient visits per year, more adverse events, higher health care costs including the prescription of multiple medications, and having a lower health-related quality of life [31–33], This can be partially attributed to the fact that the average age and Charlson index score in our study were higher amongst patients with chronic diseases. In accordance with this finding, Østbye et al. found that chronic illnesses require more time then physicians have available for patient care [34].

In an overworked primary care system, facing growing numbers of elderly and chronically ill patients as well as mounting guidelines and tests, providing the required preventive, chronic and acute medicine and maintaining high quality of care is becoming an extremely difficult task [35].

To deal with these rising challenges on current models of primary health care, other forms of care such as shared medical appointments have been suggested [36]. This model of non-physician clinicians was also suggested by Yarnall et al., who proposed another solution in the form of many more shorter visits per year [37]. Additional recommendations include comprehensive primary care guidelines that integrate highly correlated diseases together, as well as patient education [34].

## Conclusion - implications for research and/or practice

In our review, we noticed the existence of a global variety of health organizations and operative units, accompanied by an increasing workload and a growing complexity of guideline-based primary care. The various international comparisons do not take into account the variability in PCP visit duration from one country to the next as well as the differences between health care systems. This in turn results in diverse guidelines as to how to organize the schedule of PCP visits duration. We suggest that this concept of AADT may serve as a new standardized comparative measure, by facilitating the standardization of PCP’s working hours to 1000 patients and accordingly the number of allocated PCP positions required. This makes it easier to evaluate and unify the characteristics of high quality primary care. However, further research is necessary to evaluate the potential of this novel concept. ‬

Another issue to address is that of chronically-ill patients’ follow-up. Due to current time constraints and limitations, it is clear that sufficient follow up and management cannot be conducted in a single visit. Our findings support cumulative duration as a parallel indicator (to the number of visits) for quality of care, and therefore there is room to evaluate whether new PCP guidelines should also refer to the optimal amount of time needed to be spent on health topics addressed within the PCP setting, rather than focusing on the number of visits.

In our study, we found that the AADT spent with a PCP is affected by the same variables as the number of visits. This finding should be evaluated by further research, which is required to assess the benefits of new practice models dealing with the allocation of time and how well they provide quality of care in the primary setting, by relating among others AADT to clinical outcomes and other relevant quality measures.

While facing the ongoing increase in a PCP’s work-load and continuous shortening of visit length, the novel concept of AADT gives a new measure to facilitate in health care policy design, compare between different healthcare systems that allocate different time frames for a single primary care visit, and plan time-consuming tasks (such as chronic disease follow up) as well as asses their contribution in terms of ‘physician time’ vs. number of visits.

## Additional file

Additional file 1: Linear regression model of AADT (in minutes) spent with a PCP during 2012 according to age - B coeficient (Age group 20-29=1). (PDF 55 kb)

## Abbreviations

AADT: Accumulated Annual Duration of Time
CDC: Centers for Disease Control and Prevention
CHF: Chronic Heart Failure
CHS: Clalit Health Services
COPD: Chronic Obstructive Pulmonary Disease
HMO: Health Maintenance Organization
ICBS: Israel Central Bureau of Statistics
IHD: Ischemic Heart Disease
IQR: Interquartile Range
PCP: Primary Care Physician
s/p CVA: status post Cerebrovascular Accident
SES: Socioeconomic Status

## References

[1] Muggah E, Graves E, Bennett C, Manuel DG (2012). The impact of multiple chronic diseases on ambulatory care use; a population based study in Ontario, Canada. BMC Health Serv Res.

[2] Bronshtein O, Katz V, Freud T (2006). Techniques for terminating patient-physician encounters in primary care settings. Isr Med Assoc J.

[3] Petterson SM, Liaw WR, Phillips RL (2012). Projecting US primary care physician workforce needs: 2010-2025. Ann Fam Med.

[4] Schäfer W, Kroneman M, Boerma W (2010). The Netherlands: health system review. Health Syst Transit.

[5] Cherry DK, Hing E, Woodwell DA, et al. CDC National Ambulatory Medical Care Survey: 2006 Summary; Division of Health Care Statistics - National Health Statistics Reports Number 3. http://www.cdc.gov/nchs/data/nhsr/nhsr003.pdf. Accessed 6 Aug 2008. 18972720

[6] Deveugele M, Derese A, van den Brink-Muinen A (2002). Consultation length in general practice: cross sectional study in six European countries. BMJ.

[7] Bindman AB, Forrest CB, Britt H, Crampton P, Majeed A (2007). Diagnostic scope of and exposure to primary care physicians in Australia, New Zealand, and the United States: cross sectional analysis of results from three national surveys. BMJ.

[8] Blumenthal D, Causino N, Chang YC (1999). The duration of ambulatory consultations to physicians. J Fam Pract.

[9] Freeman GK, Horder JP, Howie JGR (2002). Evolving general practice consultation in Britain: issues of length and context. BMJ.

[10] Landau Y, Vinker S, Shani M, Nakar S (2008). Has the time come to adopt consultation time as a new technology for “the basket”? A literature review of the relations between consultation duration and consultation quality in primary care. Harefuah.

[11] Roland MO, Bartholomew J, Courtenay MJ (1986). The "five minute" consultation: effect of time constraint on verbal communication. BMJ.

[12] Wilson A, McDonald P, Hayes L (1992). Health promotion in the general practice consultation: a minute makes a difference. BMJ.

[13] Nowalk MP, Bardella IJ, Zimmerman RK (2004). The Physician's office: can it influence adult vaccination rates?. Am J Manag Care.

[14] Sabatino SA, Thompson T, Coughlin SS (2009). Predisposing, enabling, and reinforcing factors associated with mammography referrals in U.S. primary care practices. Open Health Serv Policy J.

[15] Weiss LJ, Blustein J (1996). Faithful patients: the effect of long-term physician-patient relationships on the costs and use of health care by older Americans. Am J Public Health.

[16] Wilson A, Childs S (2002). The relationship between consultation length, process and outcomes in general practice: a systematic review. Br J Gen Pract.

[17] Wilson AD, Childs S (2006). Effects of interventions aimed at changing the length of primary care physicians’ consultation. Cochrane Database Syst Rev.

[18] Tai-Seale M, McGuire TG, Zhang W (2007). Time allocation in primary care office consultations. Health Serv Res.

[19] Salisbury C, Procter S, Stewart K (2013). The content of general practice consultations: cross-sectional study based on video recordings. Br J Gen Pract.

[20] Abbo ED, Zhang Q, Zelder M (2008). The increasing number of clinical items addressed during the time of adult primary care consultations. J Gen Intern Med.

[21] Tsai AG, Abbo ED, Ogden LG (2011). The time burden of overweight and obesity in primary care. BMC Health Serv Res.

[22] Chen LM, Wildon RF, Ashish KJ (2009). Primary care visit duration and quality does good care take longer?. Arch Intern Med.

[23] Gottschalk A, Flocke SA (2005). Time spent in face-to-face patient care and work outside the examination room. Ann Fam Med.

[24] Charlson ME, Pompei P, Ales KL, MacKenzie CR (1987). A new method of classifying prognostic comorbidity in longitudinal studies: development and validation. J Chronic Dis.

[25] De Groot V, Beckerman H, Lankhorst GJ, Bouter LM (2003). How to measure comorbidity: a critical review of available methods. J Clin Epidemiol.

[26] Gray DJP (1998). Forty-seven minutes a year for the patient. Brit J Gen Prac.

[27] Irving G, Reeve J (2012). Do GPs really provide 47 minutes a year for the patient?. Br J Gen Pract.

[28] Hogan C, Lunney J, Gabel J, Lynn J (2001). Medicare beneficiaries' costs of care in the last year of life. Health Aff (Millwood).

[29] Riley GF, Lubitz JD (2010). Long-term trends in Medicare payments in the last year of life. Health Serv Res.

[30] Langton JM, Blanch B, Drew AK, Haas M, Ingham JM, Pearson SA (2014). Retrospective studies of end-of-life resource utilization and costs in cancer care using health administrative data: a systematic review. Palliat Med.

[31] Field TS, Gurwitz JH, Harrold LR (2004). Risk factors for adverse drug events among older adults in the ambulatory setting. J Am Geriatr Soc.

[32] Vogeli C, Shields AE, Lee TA (2007). Multiple chronic conditions: prevalence, health consequences, and implications for quality, care management, and costs. J Gen Intern Med.

[33] Wolff JL, Starfield B, Anderson G (2002). Prevalence, expenditures, and complications of multiple chronic conditions in the elderly. Arch Intern Med.

[34] Østbye T, Yarnall KSH, Krause KM (2005). Is there time for Management of Patients with Chronic Diseases in primary care?. Ann Fam Med.

[35] Discussion paper: Patient safety implications of general practice workload. Royal College of General Practitioners. 2015. Accessible at: http://www.rcgp.org.uk/policy/rcgp-policy-areas/fatigue-in-general-practice.aspx.

[36] Heyworth L, Rozenblum R, Burgess JF (2014). Influence of shared medical appointments on patient satisfaction: a retrospective 3-year study. Ann Fam Med.

[37] Yarnall KSH, Østbye T, Krause KM (2009). Family physicians as team leaders: “time” to share the care. Prev Chronic Dis CDC.

