# Supplementary figures and images for: A new marker of primary care utilization - annual accumulated duration of time of visits
Source: Isr J Health Policy Res. 2017 Aug 10;6:35. doi: 10.1186/s13584-017-0159-y (PMC5550929; doi:10.1186/s13584-017-0159-y)

## APPENDIX A

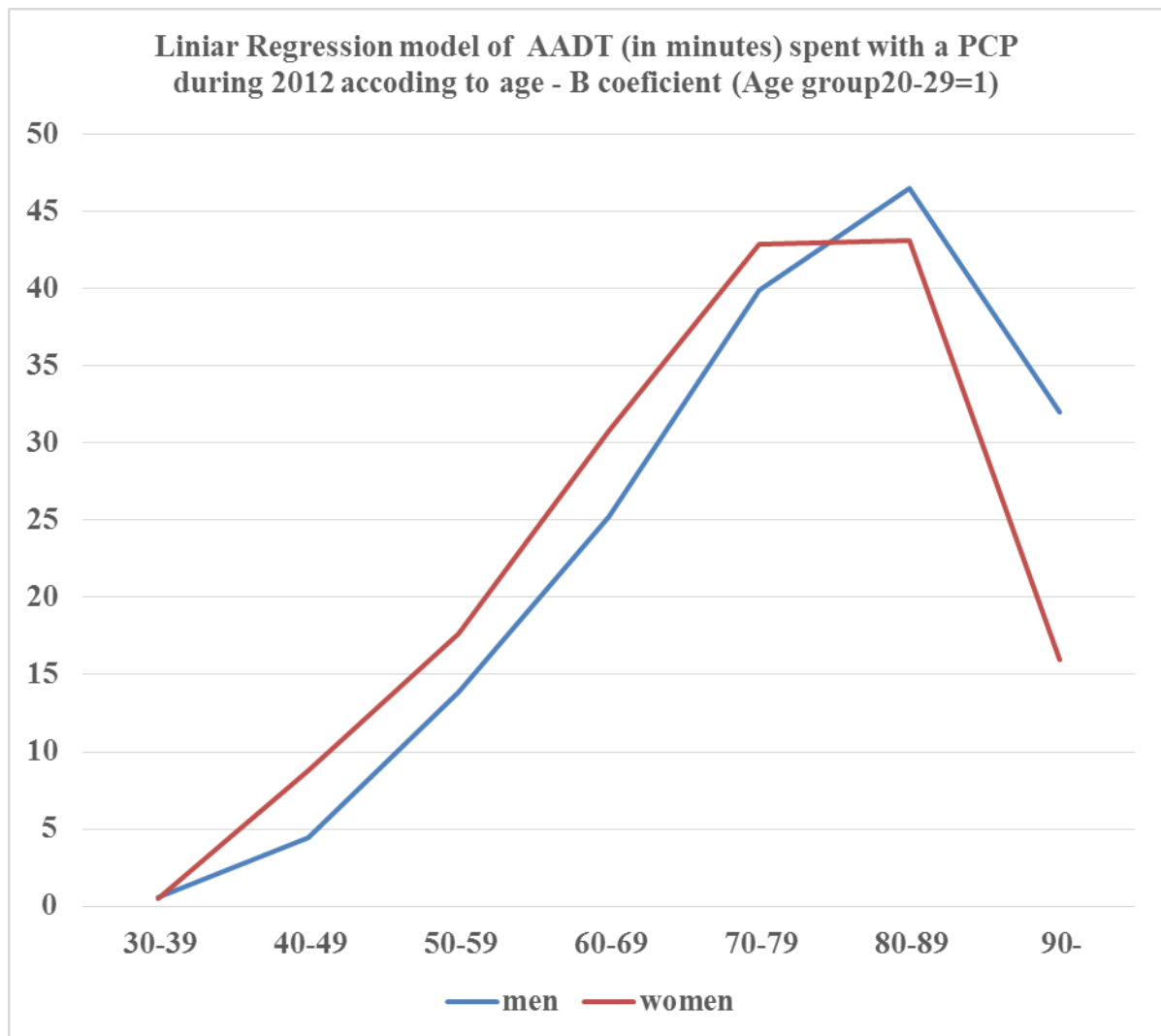

Supplement: Additional file 1: — Linear regression model of AADT (in minutes) spent with a PCP during 2012 according to age - B coeficient (Age group 20-29=1). (PDF 55 kb) [file 13584_2017_159_MOESM1_ESM.pdf]
